# Comparison of mailed invitation strategies to improve fecal occult blood test participation in men: protocol for a randomized controlled trial

**DOI:** 10.1186/1745-6215-14-239

**Published:** 2013-07-31

**Authors:** Amy Duncan, Ian Zajac, Ingrid Flight, Benjamin JR Stewart, Carlene Wilson, Deborah Turnbull

**Affiliations:** 1 School of Psychology, The University of Adelaide, Adelaide 5005, South Australia, Australia; 2 Preventative Health Flagship, Commonwealth Scientific and Industrial Research Organisation (CSIRO), Adelaide 5000, South Australia, Australia; 3 Flinders Centre for Innovation in Cancer, Flinders University, Bedford Park 5042, South Australia, Australia

**Keywords:** Colorectal neoplasm, Mass screening methods, Male, Randomized controlled trial, Protocol, Psychological factors

## Abstract

**Background:**

Men have a significantly increased risk of being diagnosed with, and dying from, colorectal cancer (CRC) than women. Men also participate in fecal occult blood test (FOBT) screening at a lower rate than women. This study will determine whether strategies that target men’s attitudes toward screening, and matched to stage of readiness to screen, increase men’s FOBT participation compared to a standard approach.

**Methods/Design:**

Eligible trial participants will be a national sample of 9,200 men aged 50 to 74 years, living in urban Australia and randomly selected from the Australian electoral roll. Trial participants will be mailed an advance notification letter, followed 2 weeks later by an invitation letter and a free fecal immunochemical test (FIT) kit. The intervention is a factorial design, randomized controlled trial (RCT) with four trial arms, including a control. The content of the advance notification and invitation letters will differ by trial arm as follows: 1) standard advance notification and standard invitation (control arm); 2) targeted advance notification and standard invitation; 3) standard advance notification and targeted invitation; and 4) targeted advance notification and targeted invitation. The standard letters will replicate as closely as possible the letters included in the Australian National Bowel Cancer Screening Program (NBCSP). Modified advance notification and invitation letters will incorporate additional messages to target men in the precontemplation (advance notification) and contemplation stages (invitation). The primary outcome is return of the completed FIT within 12 weeks of invitation. Analysts will be blinded to trial assignment and participants will be blinded to the use of varying invitational materials. Subsamples from each trial arm will complete baseline and endpoint surveys to measure the psychological impact of the intervention, and qualitative interviews will be conducted to evaluate attitudes toward the intervention.

**Discussion:**

The outcomes of this study will have implications for the way FOBT screening is offered to men. Findings will help to identify how invitations for men to screen should be framed and delivered in order to maximize participation.

**Trial registration:**

Australian New Zealand Clinical Trials Registry: ACTRN12612001122842

## Background

Australia has one of the highest incidence rates of colorectal cancer (CRC) in the world, exceeding Europe, North America and many other Western countries [1]. In Australia, there are approximately 12,000 new cases of CRC and 4,500 CRC-related deaths per annum [2]. The risk of CRC significantly increases from the age of 50 years and continues to increase with age [3]. Although CRC can occur in both men and women, men are 1.4 times more likely to be diagnosed with CRC and 1.5 times more likely to die of CRC than women [4]. Australian longitudinal data also show a 13% increase in male CRC incidence rates from 1982 to 2007, whereas female incidence rates over this period remained relatively stable [2]. These data highlight the significance of CRC as a public health problem in the aging Australian male population.

Regular screening with fecal occult blood tests (FOBTs) has been shown to significantly reduce CRC incidence and mortality by up to 25% in randomized controlled trials (RCTs) [5]. Consequently, population screening programs utilizing FOBT screening have been established in many countries, including Australia, France, Italy and the UK [6]. The National Bowel Cancer Screening Program (NBCSP) in Australia currently offers free, mail-delivered FOBT screening to Australians turning 50, 55, 60 or 65 years of age in any given year, with plans to gradually extend screening to the broader population [7]. To date, the NBCSP has offered screening to over 1,500,000 Australians [8]; however, participation in the program is suboptimal. Recent program data show that of the 685,000 people offered screening in 2008, only 40.1% completed the test [8]. Low participation in the program is problematic because it does not permit the program to achieve its full public health benefit. Given that men have a significantly higher risk of CRC than women, the finding that male participation in the program has been consistently lower than female participation is also of concern [8,9]. In 2008, for example, 36.7% of invited men completed the FOBT compared to 43.5% of women [8]. This gender disparity is consistent with FOBT screening data worldwide. Specifically, large population-based studies in the USA, Canada and Denmark have demonstrated that the participation rate of women is 1.2 to 1.8 times higher than that of men [10-15]. These data highlight the importance of identifying ways of increasing male participation in FOBT screening.

In order to identify strategies to improve FOBT participation by men, an understanding of the influences on screening behaviour is important. Several established theoretical models from the health psychology literature, including the health belief model (HBM) [16], the theory of planned behaviour (TPB) [17] and the preventive health model (PHM) [18], have been utilized to explain screening intention and behaviour, and form the basis of many behavioral interventions to improve adherence [19]. These models posit that cognitive, social and attitudinal constructs all relate to health behaviour decisions. Manipulation of these constructs is therefore required to encourage behaviour change.

Our analyses (unpublished), validated across two population data sets, found that men who perceive fewer barriers to FOBT screening, greater benefits, greater self-efficacy for completing the test, have more sources of social support who believe they should screen, and are motivated to comply with societal attitudes and beliefs in regards to screening (social influence), were more likely to complete the FOBT. These data were collected via mailed surveys from two urban, South Australian populations of men and women aged 50 to 74 years inclusive, and selected at random from the Australian electoral roll. Population characteristics and recruitment methods for these samples are reported in more detail elsewhere [20,21]. Several of these findings are supported in the wider research literature [22-24] and provide a useful starting point for the development of interventions, to target attitudes toward screening and encourage screening use in men.

Recent reviews indicate that interventions that target the needs of population subgroups are more likely to be effective than a whole population approach [25,26]. Stage theories, including the transtheoretical model (TTM) of behaviour change [27], are often utilized to describe readiness to participate in screening and have proved useful for characterizing the population in order to develop targeted interventions [28]. These theories deconstruct screening behaviour into discrete stages of readiness (intention) to participate in screening and each stage is hypothesized to be associated with different psychosocial characteristics. Forward progression through the stages is theoretically achieved by targeting the variables relevant to the stage of readiness of the target population [27]. The TTM describes five sequential stages of readiness to screen: 1) precontemplation (no intention to participate in screening); 2) contemplation (intention to participate in screening); 3) preparation (preparing to participate in screening); 4) action (participated in screening); and 5) maintenance (sustained screening participation over regular intervals).

Several studies combining stage theories with the health behaviour models mentioned previously have identified differences between participants in the earlier (precontemplation and contemplation) and later (action and maintenance) stages of readiness for a variety of psychosocial constructs. For example, participants in the earlier stages of readiness have been shown to report greater barriers to screening [28,29] and have lower perceived CRC risk [29] compared to those in the later stages who report greater health benefits of screening [28-31]. These findings suggest that the information needs of non-participants differ according to readiness to engage in screening. Interventions aiming to improve participation are therefore likely to benefit from a stage-matched approach that targets these needs.

Consistent with a number of international screening programs [32], the NBCSP invites participants to screen by the use of mailed invitation letters accompanied by a free fecal immunochemical test (FIT) [7]. A FIT is a type of FOBT that does not require dietary restrictions prior to its use [33]. The current invitation protocol for the NBCSP operates along a framework consistent with the TTM. First, participants are mailed an advance notification letter informing them that within 2 weeks they will receive a screening package. The aim of the advance notification is to encourage movement from precontemplation (not yet thinking about screening) to contemplation (thinking about screening) [34]. The subsequent invitation letter and associated screening package then seeks to shift these participants from contemplation (theoretically achieved by the advance notification) to the action stage; completing the FOBT. Although this contact schedule is consistent with the TTM staging framework, the content of the letters is not tailored according to the stage that each letter is designed to target. Including messages that target psychosocial variables relevant to men in these stages has the potential to encourage stage progression and improve screening uptake. Specifically, perceived barriers, social influence and social support all operate at the early stages of readiness, and should be emphasized in advance notification letters; while perceived benefits and self-efficacy operate at the later stages of readiness, and are best addressed in communication aimed at invitation [29-31].

The objectives of this study are to determine: 1) whether invitational strategies that target male attitudes toward screening, and matched to stage of readiness to screen, enhance FOBT participation compared to a standard approach; 2) which combination of targeted and standard materials are most effective in enhancing participation; and 3) whether targeted invitational strategies lead to change in psychosocial variables related to male screening participation. This study will also explore men’s reactions to, and evaluation/acceptance of, the invitations.

## Methods

### Study design

This study is a factorial design, RCT. A national sample of Australian men aged 50 to 74 years inclusive, will receive a free, mail delivered FIT, accompanied by either standard invitational materials (control), invitational materials modified to target psychosocial variables associated with stage of readiness to screen, or a combination of the standard and modified materials. The intervention will offer screening to participants consistent with the protocol utilized by the NBCSP, which includes a mailed advance notification letter, followed 2 weeks later by a mailed invitation letter and screening package. The content of the advance notification and invitation letters will differ by trial arm. The letters currently utilized by the NBCSP will serve as the control. The modified advance notification letter will be designed to target men in the precontemplation stage, while the modified invitation letter will target men in the contemplation stage.

Participants will be randomized to one of four trial arms of equal size (allocation ratio 1:1:1:1). Within each trial arm, participants will be randomized to a survey group, or a screening only group (allocation ratio 1:2.83). Participants in the survey group will complete behavioral surveys measuring the psychosocial variables targeted in the intervention, before commencing the screening phase (baseline) and again upon its completion (endpoint). Participants in the screening only group will receive the screening offer, without prior study engagement, consistent with the NBCSP. The use of behavioral surveys pre- and post-intervention will allow the study to examine the impact of the letters on the psychosocial variables they are designed to target. However, survey participation will be restricted to a survey subgroup because of the potential for completion of the survey to lead to increased screening awareness for this population.

**Figure 1:**
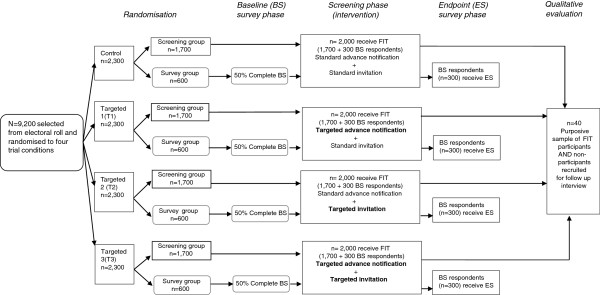
Study design.

Upon completion of the screening phase, a purposively selected subsample of participants in the screening only group will be invited to participate in semi-structured interviews to evaluate the intervention. The study design flow chart is shown in Figure 1.

### Participants

The names and addresses of 9,200 potential trial participants will be randomly selected from the Australian electoral roll. Men aged 50 to 74 years inclusive, and living in the urban regions of South Australia, Western Australia, New South Wales, Victoria and Queensland, will be eligible. The extract obtained from the electoral roll will be stratified to reflect the population density of the five Australian states.

The personal information provided by the Australian electoral commission will be used specifically for the purpose of contacting participants via mail, either to recruit participants into the survey group, or to offer a FIT in a manner equivalent to the NBCSP. Mailed invitations will include study information and contact details (mail, email and telephone) for the research team and the Human Research Ethics Committee, The University of Adelaide, Australia. Participation is voluntary and participants can opt-out of receiving further communication from the study at any time. Consent procedures for each phase of the study are described in the relevant sections.

### Randomization

Trial participants will be randomly sorted according to an assigned random integer from 1 through n (total eligible sample). Subsequently, block randomization will be performed using random allocation software [35] with the four trial conditions specified, and with random block sizes of 4, 8 and 12. Participants will then be assigned to trial conditions according to the randomly assigned integer and its corresponding trial condition specified in the block randomization output. Following allocation to trial conditions, participants will be assigned a random integer from 1 through n within each condition, and those assigned integers 1 through 600 in each condition will be assigned to the survey group for the corresponding trial arm. Randomization will be performed by a member of the research team (not the primary investigator) using de-identified data (participant names will be replaced by a numerical study identification number).

### Survey phases

Participants allocated to the survey group will be invited to complete a baseline behavioral survey prior to commencing the screening phase of the trial. Participants will receive a survey advance notification letter by mail, followed 2 weeks later by the survey package. Reminder letters will be mailed at 3 and 7 weeks to non-respondents. Data collection will cease at 12 weeks. Respondents will indicate consent to participate via return of the completed survey. Survey invitees will receive up to four letters inviting them to participate in the survey during the 12-week data collection period. No further attempts will be made to recruit non-respondents for either the survey, or the screening phase after this period on the basis that subsequent study invitations could be construed as harassment. This approach is consistent with previous population screening research utilizing electoral roll data [36]. Upon completion of the screening phase, baseline respondents will be invited to complete an endpoint survey via the same recruitment methods utilized for the baseline phase.

The baseline survey will collect demographic data (age, marital status, education, employment status, country of birth, language spoken at home and insurance status) and will obtain a baseline measurement for each of the psychosocial constructs targeted in the intervention (stage of readiness to screen, perceived barriers, benefits, social influence, social support, susceptibility and self-efficacy). Stage of readiness to screen will be measured using a staging algorithm utilized in previous research [36]. Each variable will be measured using a number of individual items and summed for an overall score. Response format for the psychosocial variables will be a five-point Likert scale ranging from ‘strongly disagree’ to ‘strongly agree’. It is anticipated that the surveys will take between 20 and 30 minutes to complete. Survey variables and example items are summarized in Table 1. The psychosocial measures and stage of readiness questions will then be repeated in the endpoint survey, in order to measure change from baseline to endpoint following exposure to the intervention.

**Table 1:** Summary of variables included in the psychosocial survey and example items

**Variable**	**Items**	**Example**	**Reference**
Perceived barriers	11	A home stool test is embarrassing	Rawl *et al*. (2001) [37]
Perceived benefits	5	A home stool test can find cancer early	Rawl *et al*. (2001) [37]
Self-efficacy	5	I am confident that I would be able to screen for bowel cancer using a home stool test even if I find the test to be embarrassing	Duncan *et al.* (2012) [20]
Susceptibility	4	Compared with other persons my age, I am at higher risk for bowel cancer	Flight *et al.* (2010) [38]
Social influence	4	I want to do what my doctor thinks I should do about bowel cancer screening	Flight *et al.* (2010) [38]
Social support	11	There is someone I can talk to about the pressures in my life	Shakespeare-Finch *et al.* (2011) [39]

### Intervention (screening phase)

The intervention involves four arms: 1) standard advance notification letter and standard invitation letter consistent with the NBCSP (control); 2) targeted advance notification letter plus standard invitation letter (T1); 3) standard advance notification letter plus targeted invitation letter (T2); and 4) targeted advance notification letter and targeted invitation letter (T3).

Screening will be offered to all baseline survey respondents and participants in the screening only groups according to the schedule described in Table 2.

**Table 2:** Screening schedule

**Week**	**Study process**
Week 1	Mailed advance notification
Week 3	Mailed invitation and screening package
Week 9	Mailed reminder letter to non-respondents
Week 15	Cease data collection

Screening packages will include: 1) two-sample FIT (OC-Sensor, Eiken Chemical Co, Tokyo, Japan); 2) screening invitation letter; 3) FIT instruction leaflet; 4) CRC and screening information booklet; 5) participant details and consent form; and 6) reply paid envelope. Those who do not participate in the FIT screening offer within the first 6 weeks will receive a standard reminder letter irrespective of trial allocation. Consent to participate in the screening will be obtained via return of the completed participant details and consent form, and/or return of the completed FIT.

It is anticipated that some participants invited to screen will already be up to date with screening or may not be suitable for screening due to current medical conditions (for example chronic bowel conditions and past CRC diagnosis [40]). Consistent with the NBCSP, participants will be encouraged to first discuss screening with their general practitioner and to then contact the research team if screening is not required. Reasons for non-participation (for example up to date with screening, medical reason and no reason provided) will be recorded for analysis. A screening withdrawal form, where invitees can provide reasons for non-participation, will be included in the screening information booklet to assist with this process, along with telephone and email contact information.

### Letter content

The targeted advance notification and invitation letters included in the intervention will be based on the standard letters utilized by the NBCSP and modified according to best practice guidelines [41-44]. In order to determine whether the inclusion of targeted material improves screening outcomes over and above the standard NBCSP information, the modified letters will retain the content of the control letters in addition to the targeted messages. Targeted letters will also include changes to formatting (for example the use of subheadings) consistent with best practice guidelines and to assist with the organization of the expanded material [41]. All additional screening material (for example participant details form and screening information booklet) will be designed to mimic as closely as possible the materials provided in the NBCSP [7]. The following sections describe each of the intervention letters in more detail.

#### Advance notification letter

The standard advance notification letter (control) briefly introduces FIT screening and its purpose, informs participants that a FIT package will be mailed to them, and describes how personal details were obtained and will be protected. The targeted intervention letter will build on this standard letter by incorporating additional messages designed to encourage movement from precontemplation to contemplation. Specifically, messages will aim to reduce common barriers to screening (for example ‘the FOBT is designed to be simple and hygienic’), encourage discussions about screening with sources of social support and influence (for example ‘if you have any questions about screening you should talk to your doctor. It may also be helpful to discuss screening with a family member or a close friend’) and emphasize CRC susceptibility among men aged 50 and older (for example ‘although both men and women can get bowel cancer, it is more likely to occur in men’).

#### Screening invitation letter

The standard invitation letter summarizes the content of the invitation package, emphasizes the importance of reading the screening materials provided, and briefly describes how FIT results will be processed and returned. The targeted invitation will include messages designed to encourage movement from contemplation to action. This letter will include messages designed to improve perceptions of the benefits of participating in the FIT (for example ‘screening for bowel cancer using a fecal occult blood test can reduce the chances of dying from bowel cancer’) and to increase self-efficacy for completing the test (for example ‘the step-by-step instructions will help you to complete the test even if you have never done a test like this before’). As with the advance notification, susceptibility to CRC among men will also be emphasized.

### Qualitative evaluation

Following the screening phase, a purposively selected group of participants from the screening only groups will be invited to take part in semi-structured telephone interviews to explore men’s reactions to, and satisfaction with, the intervention. Participants invited to qualitative interviews will be selected utilizing maximum variation sampling to ensure the subsample varies by age, intervention group and participation in the screening offer. Invitations will be sent by mail, and will include an opt-in form, study information, research team and HREC contact details, and a reply paid envelope. Those wishing to participate in the interviews will be asked to contact the research team (by telephone, email or return of the opt-in form) to indicate willingness to be involved in the interviews. Verbal consent will be obtained prior to the interview.

It is estimated that the qualitative interviews will last between 15 and 30 minutes and will be conducted one-on-one using computer assisted telephone interviewing (CATI). Questions will focus on: 1) why participants chose or chose not to use the FIT; 2) reactions to, and satisfaction with, the intervention; and 3) how participants feel the intervention materials could be improved. Interviews will be audio recorded and transcribed prior to analysis.

### Outcomes

The primary outcome will be participation in the screening test, defined as return of the completed FIT within 12 weeks of the invitation. Secondary outcomes (stage of change, psychosocial variables and qualitative evaluation) will be collected via the baseline and endpoint surveys, and the qualitative evaluation. The data collected during each phase is summarized in Table 3.

**Table 3:** Data collection phases and measures obtained

**Phase**	**Data collection**	**Measures**
Phase 1	Baseline survey mailed to psychosocial subgroup (n = 2,400); reminders sent 3 and 7 weeks from initial mailing	Demographic characteristics: age, education, country of birth, marital status, employment, insurance coverage.
		TTM stage: decision stage for screening assessed by TTM stage (precontemplation, contemplation, action, maintenance).
		Psychosocial constructs: barriers, benefits, self-efficacy, susceptibility, social influence and social support.
Phase 2	Screening advance notification and invitations; reminders sent 6 weeks after mailing of invitation	Receipt or non-receipt of completed FIT, date of return of FIT, reasons for opt-out recorded by the Bowel Health Service, Repatriation General Hospital, Adelaide, Australia.
Phase 3	Endpoint survey mailed to baseline survey respondents (n = 1,200, estimated); reminders sent 3 and 7 weeks from initial mailing	TTM stage and psychosocial constructs as measured in the baseline survey.
Phase 4	Qualitative evaluation; data obtained following screening phase from focus groups, including participants from each of the trial arms	Why participants chose or chose not to use the FIT.
		Reaction to, and satisfaction with, the intervention.
		How intervention materials could be improved.

*FIT*, fecal immunochemical test; *TTM*, transtheoretical model.

### Sample size considerations

Assuming a 50% return of the baseline survey, screening will be offered to a total of 8,000 participants (1,200 survey participants and 6,800 screening only participants). Participants who contact the research team and opt-out of screening due to: 1) recent FOBT screening; 2) recent colonoscopy; 3) recent bowel cancer diagnosis; or 4) medical condition that prevents the use of FOB-based screening tests, will be excluded from analysis on the basis that screening is not suitable/required for this group [3]. Those who opt-out of screening for reasons other than those listed previously (for example did not want to complete the screening) will be categorized as a non-participant for the purpose of analyses. Survey group participants who opt-out of the study prior to completing the endpoint phase will be excluded from analyses involving survey data.

Two participation rates will then be calculated for each trial arm: 1) inclusive of survey participants (2,000 per trial arm); and 2) exclusive of survey participants (1,700 per trial arm). Analyses will be conducted both with and without the inclusion of survey participants because participation for this subgroup is likely to be higher due to the baseline survey acting as a prompt for screening participation. Assuming a participation rate of at least 40% for the control group and allowing for up to 40% of invited participants to be excluded due to being up to date with screening [8], these group sizes permit the detection of differential FOBT uptake of at least 6% between any two groups with at least 80% power. A difference of at least 6% was considered by the research team to be the smallest difference that would be feasible to detect without requiring overly large sample sizes, and is similar to the effect size estimate used in our previous research [36].

### Blinding

Data collectors and analysts will be blinded to trial allocation following initial randomization. Data analysts will become unblinded following analysis of the primary outcome for reporting purposes. All trial participants will receive an invitation to screen for free as part of a large male health study, but will remain blinded to the use of varying invitational techniques across trial conditions.

### Statistical analyses

#### Primary outcome

A 2 x 4 (participation by intervention group) chi-squared test with alpha set at 0.05 will compare FIT participation rates between groups. Potential covariates such as demographic factors (age and socioeconomic status) and psychosocial variables (survey sample only) will be checked by the change in coefficient method using log binomial generalized linear models.

#### Secondary outcomes

Change in psychosocial variables from baseline to endpoint will be calculated for the participants who complete both surveys. Change scores will be calculated by subtracting baseline from endpoint scores for each of the variables targeted by the intervention. These change scores will then be compared between intervention groups using analysis of covariance (ANCOVA), controlling for potential co-variation from demographic variables. Based on the predicted group sizes mentioned above, the ANCOVA using two-tailed significance will have 80% power to detect a difference of at least half a standard deviation for psychosocial variables between any two groups.

Qualitative data collected via telephone interviews will be analyzed using the framework analysis method recommended for applied health research by Green and Thorogood [45]. This method is suited to applied health research as it allows for the exploration of themes derived from *a priori* aims relevant to this research stage as well as emergent themes raised by respondents. The study will aim to recruit 20 screening participants and 20 non-participants for interview in order to achieve data saturation [45].

### Ethical considerations

This trial has been approved by the Human Research Ethics Committee, The University of Adelaide, Australia.

## Discussion

This project will identify whether recruitment strategies targeted to stage of readiness to screen, and the variables known to influence men’s movement to a higher stage, are effective in encouraging men to participate in FIT screening. The outcomes will provide information concerning how messages can be framed and delivered, in order to maximize participation in population screening programs in Australia and globally. If this study shows that a stage-matched intervention that addresses male concerns improves participation, the information may be useful for optimizing male participation in alternative forms of screening and preventive health practices. Future research incorporating a female population would be required to establish whether differences in participation result from the gendered information, or are a consequence of the overall improvements made to the screening invitation letters.

This study is among the first to dedicate a specific focus to understanding male screening behaviour. While several national screening initiatives exist for women in Australia (for example breast and cervical cancer screening programs) the NBCSP is the first nationally funded screening program to include men [7]. This study will collect extensive data on male attitudes toward screening through the use of quantitative surveys and qualitative interviews, and the results may also have important implications for future interventions.

A potential limitation of the study is that while the intervention aims to replicate the invitation protocol of the NBCSP, it will not be undertaken in collaboration with the national program. It is therefore likely that some of the participants invited to screen in this study may have recently participated, or are about to participate, in the NBCSP. To account for this potential limitation, participants will be encouraged to contact the research team when participation in other screening programs is the reason for declining the study offer. We acknowledge that this approach also has its limitations because it requires participants to actively volunteer the information to the research team, and because self-reported screening adherence can be inaccurate [46]. Consequently, participation rates observed in this study may be smaller than would occur for a population not exposed to other screening programs and will be considered in the reporting of results.

## Trial status

Surveys were mailed to individuals in the survey subgroup from 1 October 2012 and data collection for this phase was completed in January 2013. Screening was offered to baseline survey participants and remaining trial participants in March 2013. This phase lasted for 3 months and concluded in June 2013. Data collection for the endpoint survey and recruitment of trial participants to the qualitative group will occur simultaneously upon completion of the screening phase. It is anticipated that all data will be collected by September 2013.

## Abbreviations

ANCOVA: Analysis of covariance; CATI: Computer assisted telephone interviewing; CRC: Colorectal cancer; FIT: Fecal immunochemical test; FOBT: Fecal occult blood test; HBM: Health belief model; NBCSP: National bowel cancer screening program; PHM: Preventive health model; RCT: Randomized controlled trial; TPB: Theory of planned behaviour; TTM: Transtheoretical model.

## Competing interests

All authors declare that they have no competing interests.

## Authors’ contributions

AD developed the intervention materials, contributed to the intervention design and participant recruitment, and drafted the manuscript. IZ, DT, IF and CW were responsible for the conception and design of the intervention, and also made substantial contributions to the design of the intervention materials, recruitment and the drafting of the manuscript. BS contributed to the conception and design of the intervention, and the drafting of the manuscript. All authors read and approved the final manuscript.
